# Neurochemical Characterization of Zinc Transporter 3-Like Immunoreactive (ZnT3^+^) Neurons in the Intramural Ganglia of the Porcine Duodenum

**DOI:** 10.1007/s12031-012-9855-9

**Published:** 2012-07-12

**Authors:** Joanna Wojtkiewicz, Sławomir Gonkowski, Maciej Równiak, Robert Crayton, Mariusz Majewski, Marek Jałyński

**Affiliations:** 1 Department of Neurology and Neurosurgery, Stem Cell Research Laboratory, Faculty of Medical Sciences, University of Warmia and Mazury, ul. Warszawska 30, 10-082 Olsztyn, Poland; 2 Department of Clinical Physiology, Faculty of Veterinary Medicine, University of Warmia and Mazury, Olsztyn, Poland; 3 Department of Comparative Anatomy, Faculty of Biology, University of Warmia and Mazury, Olsztyn, Poland; 4 Department and Clinic of Urology, Faculty of Medical Sciences, Medical University of Warsaw, Warsaw, Poland; 5 Department of Human Physiology, Faculty of Medical Sciences, University of Warmia and Mazury, Olsztyn, Poland; 6 Department of Surgery and Radiology, Faculty of Veterinary Medicine, University of Warmia and Mazury, Olsztyn, Poland

**Keywords:** Zinc-enriched neurons, Duodenum, Small intestine, Domestic pig, Immunolabeling technique, Enteric nervous system

## Abstract

The SLC30 family of divalent cation transporters is thought to be involved in the transport of zinc in a variety of cellular pathways. Zinc transporter 3 (ZnT3) is involved in the transport of zinc into synaptic vesicles or intracellular organelles. As the presence of ZnT3 immunoreactive neurons has recently been reported in both the central and peripheral nervous systems of the rat, the present study was aimed at disclosing the presence of a zinc-enriched neuron enteric population in the porcine duodenum to establish a preliminary insight into their neurochemical coding. Double- and triple-immunofluorescence labeling of the porcine duodenum for ZnT3 with the pan-neuronal marker (PGP 9.5), substance P, somatostatin, vasoactive intestinal peptide (VIP), nitric oxide synthase (NOS), leu-enkephalin, vesicular acetylcholine transporter (VAChT), neuropeptide Y, galanin (GAL), and calcitonin gene-related peptide were performed. Immunohistochemistry revealed that approximately 35, 43, and 48 % of all PGP9.5-postive neurons in the myenteric (MP), outer submucous (OSP), and inner submucous (ISP) plexuses, respectively, of the porcine duodenum were simultaneously ZnT3^+^. In the present study, ZnT3^+^ neurons coexpressed a broad spectrum of active substances, but co-localization patterns unique to the plexus were studied. In the ISP, all ZnT3^+^ neurons were VAChT positive, and the largest populations among these cells formed ZnT3^+^/VAChT^+^/GAL^+^ and ZnT3^+^/VAChT^+^/VIP^+^ cells. In the OSP and MP, the numbers of ZnT3^+^/VAChT^+^ neurons were two times smaller, and substantial subpopulations of ZnT3^+^ neurons in both these plexuses formed ZnT3^+^/NOS^+^ cells. The large population of ZnT3^+^ neurons in the porcine duodenum and a broad spectrum of active substances which co-localize with this peptide suggest that ZnT3 takes part in the regulation of various processes in the gut both in normal physiology and during pathological processes.

## Introduction

It is well known that local intestinal activity, such as muscle contraction and relaxation or mucosal ion secretion, is controlled by the enteric nervous system (ENS). The different functions of the ENS are controlled by more than 20 classes of neurons, each with a diverse variety of co-localized and co-released neurotransmitters that are unique to their target cell receptors (Brown and Timmermans 2004; Furness 2000). The porcine ENS displays considerable similarities to that of humans in respect of anatomical, physiological, and pathological characteristics, and it is organized into three layers called plexuses: myenteric plexus (MP), outer submucous plexus (OSP), and inner submucous plexus (ISP). The MP is located between the longitudinal and circular muscle layers, and the majority of these myenteric neurons takes part in the control of digestive motility (Huizinga et al. 2011), but a small subpopulation of these neurons supplies the submucosal plexuses and/or regulates the secretory functions of the gut (Brehmer et al. 1994). Neurons of the OSP (located near the internal part of the circular muscle layer) and ISP (located between the muscularis mucosa and lamina propria) generally supply the mucosal and submucosal layers and regulate the secretion and intrinsic sensory pathways of the gut in response to luminal contents as well as innervate submucosal blood vessels (Brehmer et al. 2010). However, some neurons, especially in the OSP, can also supply the circular muscle layer of the gut (Scheuermann and Timmermans 1993).

As enteric neurons usually coexpress several active substances that regulate or modulate various cellular processors of the target cell, the chemical coding of the innervating neurons is associated with the functions of the target organ (Brookes 2001; Costa et al. 1996; Furness 2000). Zinc transporter 3 (ZnT3) has been observed in the ENS of the human and porcine species (Gonkowski et al. 2007, 2009). ZnT3 is a member of the SLC 30 family of zinc transporters, which transports zinc ions from the cytoplasm into synaptic vesicles, intracellular organelles, or to the outside of the cell (Palmiter and Huang 2004). However, the role of the Zn transporter in the function of ENS is currently unknown.

The human-ZnT3 protein has 388 amino acids and is predicted to contain six transmembrane domains that form a pore lined with a histidine-rich loop (Gaither and Eide 2001). ZnT3 has been studied in zinc-enriched nerve (ZEN) terminals within the hippocampus, amygdala, neocortex, spinal cord, and superior cervical ganglion neurons (Jo et al. 2000; Wang et al. 2003; Wenzel et al. 1997). The presence of ZnT3 in different regions of the central and peripheral nervous system (Danscher et al. 2003; Wang et al. 2003; Wenzel et al. 1997) suggests that this protein, which is responsible for transporting zinc ions into synaptic vesicles, can thus be used as a marker for tracing ZEN neuronal pathways. Moreover, previous investigations suggest that ZnT3 can play an important role during pathological processes, both within the central nervous system (Frederickson et al. 2000, 2005) and in the ENS (Gonkowski et al. 2007).

Until now, only two studies report the presence of ZnT3 in the ENS of the human (Gonkowski et al. 2009) and the porcine species (Gonkowski et al. 2007). Therefore, the aim of the present study was to investigate for the first time the distribution pattern of ZnT3-immunoreactive (ZnT3^+^) neurons within the ENS and to quantitatively evaluate the number of ZnT3^+^ neurons and their chemical coding in the intramural ganglia of the porcine duodenum. Thus, the present study constitutes the introduction to further investigations that should focus on establishing the role of ZnT3 and ZEN nerve functions within the gastrointestinal tract.

## Materials and Methods

### Study Subjects

Six juvenile and clinically healthy female pigs of the Large White Polish breed (approximately 8 weeks old and 12–15 kg of body weight) were used in the present investigation. The animals were obtained from a commercial fattening farm in Poland. All animals were housed and treated in accordance with rules approved by the ethics committee (conforming to Principles of Laboratory Animal Care, NIH publication no. 86–23, revised 1985). All experimental procedures were approved by the Local Ethics Commission of the University of Warmia and Mazury in Olsztyn (no. 27/2009).

### Anesthesia and Surgery

All animals were sedated 30 min prior to the main anesthetic with atropine sulfate (Polfa, Poland; 0.04 mg/kg body weight, s.c.) and azaperone (Stressnil, Janssen Pharmaceutica, Belgium; 2.0 mg/kg body weight, i.m.). All animals were anesthetized with thiobarbital and euthanized by an overdose of the same agent (Thiopental, Sandoz, Austria; 20 mg/kg body weight, i.v.). All animals were then perfused transcardially with 4 % buffered paraformaldehyde (pH 7.4). Following fixative perfusion, small tissue blocks comprising of the duodenum (1 cm from the pylorus) were collected from all studied animals and postfixed by immersion in the same fixative for 4 h, washed twice in 0.1 M phosphate buffer (pH = 7.4, 4 °C) for 3 days, and then stored in 18 % sucrose at 4 °C until sectioning.

### Immunofluorescence Experiments

Ten-micrometer-thick cryostat sections of the duodenum samples were processed for routine double- and triple-immunofluorescence labeling using primary antisera raised in different species and species-specific secondary antibodies (Table 1). Frozen sections were removed from the freezer and dried at room temperature for 20 min, rinsed three times in phosphate-buffered saline (PBS), and incubated in a humidified chamber with a blocking buffer (0.1 M PBS, 10 % normal horse serum, 0.01 % bovine serum albumin, 1 % Tween, 0.05 % thimerosal, 0.01 % NaN_3_) for 1 h. The sections were then rinsed again in PBS and incubated overnight at room temperature with the mixture of primary antibodies using the appropriate combination of antisera: ZnT3 and pan-neuronal marker (PGP 9.5), substance P (SP), somatostatin (SOM), vasoactive intestinal peptide (VIP), nitric oxide synthase (NOS), leu-enkephalin (LENK), vesicular acetylcholine transporter (VAChT), neuropeptide Y (NPY), galanin (GAL), and calcitonin gene-related peptide (CGRP) (Table 1). After the incubation with the primary antibodies, the sections were rinsed in PBS and incubated for 1 h with biotinylated secondary antibodies (during double-labeling immunofluorescence) or with biotinylated 7-amino-4-methylcoumarin-3-acetic acid (AMCA) (during triple-labeling immunofluorescence) (Table 1). After 1 h, the sections were incubated with the mixture of fluorescein isothiocyanate (FITC) and CY3-conjugated streptavidin (Table 1), rinsed again in PBS, then mounted with carbonate-buffered glycerol (pH 8.6) and coverslipped.

**Table 1:** Specification of immune reagents

Primary antibody				
Antisera	Code	Host species	Dilution	Supplier
PGP9.5	7863-2004	Mouse	1:2,000	Biogenesis Inc, UK; www.biogenesis.co.uk
ZnT3	–	Rabbit	1:600	Gift prof. Palmiter, USA
NOS	N2280	Mouse	1:2,000	Sigma, USA; www.sigma-aldrich.com
VIP	9535-0504	Mouse	1:2,000	Biogenesis Inc, UK; www.biogenesis.co.uk
SP	8450-0505	Rat	1:300	Biogenesis Inc, UK; www.biogenesis.co.uk
SOM	8330-0009	Rat	1:100	Biogenesis Inc, UK; www.biogenesis.co.uk
LENK	4140-0355	Mouse	1:1,000	Biogenesis Inc, UK; www.biogenesis.co.uk
VAChT	H-V007	Goat	1:2,000	Phoenix, Pharmaceuticals, Inc, USA; www.phoenixpeptide.com
NPY	NZ1115	Rat	1:300	Biomol Research Laboratories Inc, USA
GAL	T-5036	Guinea pig	1:1,000	Peninsula Labs, USA; see Bachem AG; www.bachem.com
CGRP	T-5027	Guinea pig	1:1,000	Peninsula Labs, USA; see Bachem AG; www.bachem.com
Secondary antibodies				
Reagent			Dilution	Supplier
FITC–conjugated donkey–anti-mouse IgG (H + L)			1:800	Jackson, 715-095-151
FITC–conjugated donkey–anti-rat IgG (H + L)			1:800	Jackson, 712-095-153
FITC–conjugated donkey–anti-guinea pig IgG (H + L)			1:1,000	Jackson, 706-095-148
FITC–conjugated donkey–anti-goat IgG (H + L)			1:1,000	Jackson, 705-096-147
Biotinylated goat anti-rabbit immunoglobulins			1:1,000	DAKO, E 0432
Biotin conjugated F(ab)′ fragment of affinity purified anti-rabbit IgG (H + L)			1:1,000	BioTrend, 711-1622
AMCA–conjugated donkey–anti-mouse IgG (H + L)			1:50	Jackson, 715-155-151
AMCA–conjugated donkey–anti-rat IgG (H + L)			1:50	Jackson, 715-155-153
AMCA–conjugated donkey–anti-goat IgG (H + L)			1:50	Jackson, 705-156-147
CY3–conjugated streptavidin			1:9,000	Jackson, 016-160-084

### Controls

**Fig. 1:**
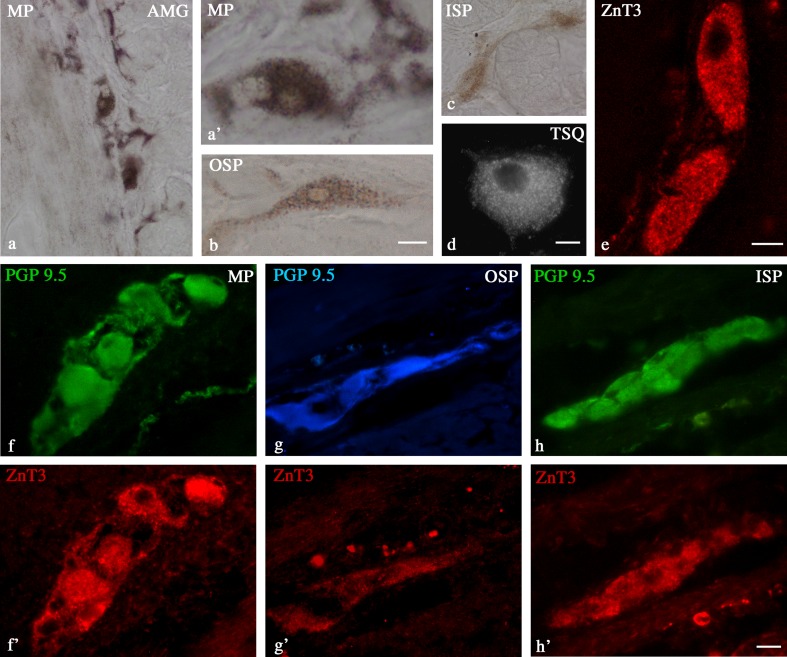
Control staining demonstrating duodenal neurons in the MP (**a**, **a′**), OSP (**b**), and ISP (**c**) stained with zinc selenide AMG and TSQ methods (**d**). Some neurons show intensity of AMG staining in the cytoplasm; High-magnification image of two ZnT3^+^ neurons (**e**); neurons in the MP, OSP, and ISP of the porcine duodenum immunostained for PGP 9.5 (**f**, **g**, **h**) and ZnT3 (**f**′, **g**′, **h**′). *Scale bars* = 100 μm (**a**), 15 μm (**a′**,**b**, **c**, **d**), 10 μm (**e**), and 25 μm (**f**–**h**′)

Standard controls, i.e., preabsorption for the neuropeptide antisera (20 mg of appropriate antigen per 1 ml of corresponding antibody at working dilution) and the omission and replacement of all primary antisera by non-immune sera or PBS, were applied to test both antibody and method specificity. To show that ZnT3 found in the enteric system is functional as a Zn transporter, the presence of free or loosely bound zinc ions in enteric neurons was tested using 6-methoxy 8-para toluene sulfonamide quinoline (TSQ) fluorescence and zinc autometallography (AMG) (Fig. 1a-d). Both the TSQ and AMG methods were conducted to show the presence of Zn in the control samples, and they were performed according to instructions described by Wang et al. (2003), Frederickson et al. (1987), and Danscher (1982).

### Counts and Statistics

The sections were viewed under an Olympus BX51 fluorescence microscope equipped with epifluorescence and appropriate set of filters for FITC, CY3, and AMCA. Microphotographs were acquired with a CCD camera connected by a PC equipped with Olympus image analysis software (ver. 3.2; Soft Imaging System GmbH, Münster, Germany). To determine the percentages of ZnT3^+^ neurons in each plexus studied (MP, OSP, and ISP), the pan-neuronal marker PGP-9.5 was adopted. PGP-9.5 marks all neurons in the tissue so PGP^+^/ZnT3^+^ cells illustrate the percentages of MP, OSP, and ISP neurons coexpressing ZnT3. At least 1,000 PGP-9.5-labeled cell bodies located in 60–70 ganglia of a particular plexus (MP, OSP, and ISP) per animal were examined for ZnT3 immunoreactivity. Only neurons with clearly visible nuclei were counted. To prevent double counting of ZnT3^+^ neurons, serial sections 200 μm apart were used. Moreover, to determine the percentages of co-localization of ZnT3 with other substances studied, at least 700 ZnT3-positive cell bodies of the same enteric plexus were examined for immunoreactivity to the particular substance investigated. In these double- and triple-labeling studies, ZnT3-positive neurons were considered as 100 % for all combinations, so all the values shown in the text and Table 2 are percentages of ZnT3^+^ neurons. These data were pooled from all six animals, expressed as means ± standard error of mean (SEM), and then analyzed by using GraphPad Prism 5 software (GraphPad Software, La Jolla, CA, USA).

**Table 2:** Neurochemical characterization of zinc transporter 3-immunoreactive (ZnT3^**+**^) neurons in the enteric ganglia of the porcine duodenum

	MP	OSP	ISP
PGP^+^/ZnT3^+^	35.3 ± 8.1	43.0 ± 9.0	48.2 ± 13.9
ZnT3^+^/VAChT^−^	60.7 ± 1.8	43.1 ± 5.4	0
ZnT3^+^/VAChT^−^/NOS^+^	29.7 ± 3.5	19.1 ± 4.8	0
ZnT3^+^/VAChT^−^/VIP^+^	4.4 ± 1.2	4.7 ± 1.6	0
ZnT3^+^/VAChT^−^/SOM^+^	4.3 ± 0.4	10.5 ± 3.1	0
ZnT3^+^/VAChT^−^/SP^+^	2.7 ± 1.51	3.9 ± 2.5	0
ZnT3^+^/VAChT^−^/LENK^+^	0.4 ± 0.5	0.4 ± 0.2	0
ZnT3^+^/LENK^+^/SP^+^	s	s	0
ZnT3^+^/VAChT^−^/GAL^+^	0	s	0
ZnT3^+^/VAChT^−^/NPY^+^	s	0	0
ZnT3^+^/VAChT^−^/CGRP^+^	0	0	0
ZnT3^+^/VAChT^+^	40.3 ± 2.9	56.9 ± 6.4	100
ZnT3^+^/VAChT^+^/SOM^+^	2.8 ± 0.8	7.4 ± 4.1	13.2 ± 3.8
ZnT3^+^/VAChT^+^/VIP^+^	2.0 ± 1.8	3.7 ± 1.4	36.6 ± 4.2
ZnT3^+^/VAChT^+^/SP^+^	1.3 ± 1.0	4.0 ± 1.7	26.2 ± 2.9
ZnT3^+^/VAChT^+^/GAL^+^	0	0	42.5 ± 1.5
ZnT3^+^/VAChT^+^/NOS^+^	0	0	0

Percentages (%) were expressed as mean ± standard error of mean (SEM). Note that PGP 9.5 is a pan-neuronal marker which marks all neurons in the tissue, so PGP^+^/ZnT3^+^ cells illustrate the percentages of MP, OSP, and ISP neurons coexpressing ZnT3. In the triple-labeling studies, ZnT3-positive neurons were considered as 100 % for all combinations with other neurotransmitters, so all the values presented in the table are percentages of ZnT3^+^ neurons

*MP* myenteric plexus, *OSP* outer submucosal plexus, *ISP* inner submucosal plexus, *s* single neurons

## Results

### The Distribution and Number of ZnT3^+^ Neurons in the Porcine Duodenum

During the present investigation, ZnT3^+^ cell bodies were observed in all types of enteric plexuses within the porcine duodenum, i.e., in the MP, located between the longitudinal and circular muscle layers; OSP, found in the submucosa; and ISP, located between the muscularis mucosa and lamina propria. The numbers of ZnT3^+^ neurons were dependent on the plexus studied. The most numerous were found within the ISP (48.2 ± 13.9 %) (Table 2 and Fig. 1h–h′). In the OSP and MP, these neurons constituted 43.0 ± 9.0 and 35.3 ± 8.1 %, respectively (Table 2, Fig. 1f–f′and g–g′). The number of ZnT3^+^ cells in the single ganglion was 2–5, 2–3, and 5–12 within the MP, OSP, and ISP, respectively. ZnT3^+^ nerve fibers were not observed in the porcine duodenum.

### The Co-localization Pattern of ZnT3^+^ Neurons in the Porcine Duodenum

In all kinds of duodenal plexuses, the population of ZnT3^+^ neurons could be subdivided into non-cholinergic (ZnT3^+^/VAChT^−^) and cholinergic (ZnT3^+^/VAChT^+^) neurons. Both non-cholinergic and cholinergic ZnT3^+^ neurons coexpressed a broad spectrum of active substances tested in the present study, but co-localization patterns varied depending on the plexus studied (Table 2).

### Myenteric Plexus

**Fig. 2:**
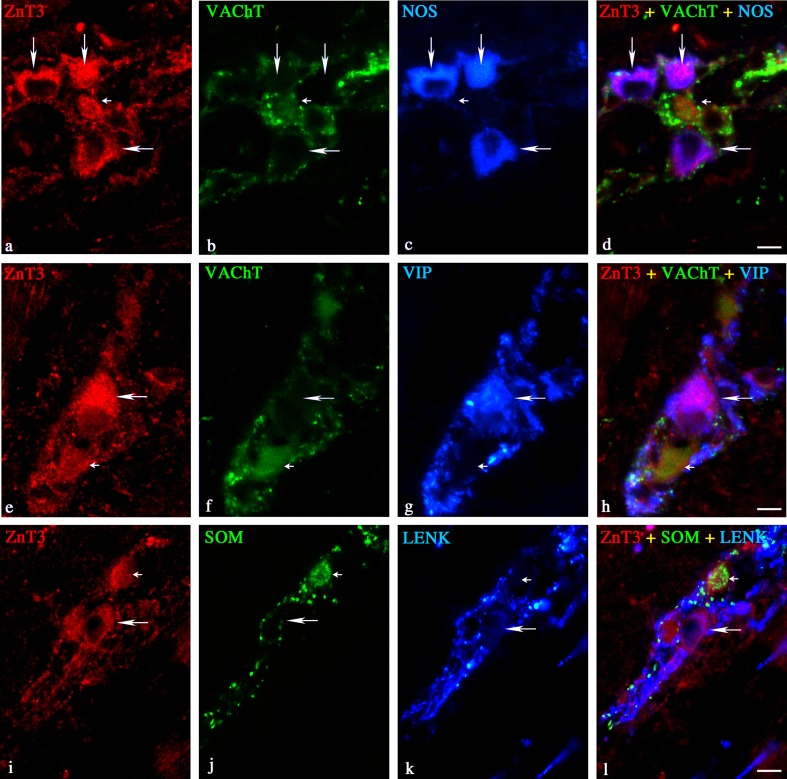
Representative images of ZnT3^+^ neurons located in the duodenal MP of the pig. Merged images (**d**, **h**, **l**) are composites of images taken separately from *red* (**a**, **c**, **i**), *green* (**b**, **f**, **j**), and *blue* (**c**, **g**, **k**) fluorescent channels. *Scale bar* = 25 μm. **a**–**d** ZnT3^+^/VAChT^−^/NOS^+^—*long arrows*, ZnT3^+^/VAChT^+^/NOS^−^—*small arrow*; **e**–**h** ZnT3^+^/VAChT^−^/VIP^+^—*long arrow*, ZnT3^+^/VACHT^+^/VIP^−^—small arrow; **i**–**l** ZnT3^+^/SOM^−^/LENK^+^—*long arrow*, ZnT3^+^/SOM^+^/LENK^−^—*small arrow*

In the MP, the non-cholinergic and cholinergic ZnT3^+^ neurons constituted 60.7 ± 1.8 and 40.7 ± 2.9 %, respectively (Table 2). Among the non-cholinergic ZnT3^+^ neurons, 29.7 ± 3.5 % were also immunoreactive to NOS (Table 2 and Fig. 2a–d). These ZnT3^+^/NOS^+^ cells were irregularly dispersed, or they were observed in groups composed of between two and five cells. In addition, 4.3 ± 0.4, 4.4 ± 1.2, 2.7 ± 1.5, and 0.4 ± 0.2 % of the non-cholinergic ZnT3^+^ neurons were also immunoreactive to SOM (Table 2 and Fig. 2i–l), VIP (Table 2 and Fig. 2e–h), SP (Table 2), and/or LENK (Table 2 and Fig. 2i–l), respectively. Only single ZnT3^+^/GAL^+^ and ZnT3^+^/NPY^+^ neurons were observed in the porcine duodenal MP while GAL and CGRP coexpression was not observed in the non-cholinergic ZnT3^+^ myenteric neurons (Table 2). Among the cholinergic ZnT3^+^ neurons, 2.8 ± 0.8 % were also immunoreactive SOM (Table 2), 2.0 ± 0.9 % were VIP positive (Table 2), and 1.3 ± 0.6 % were SP positive (Table 2). No immunoreactivity to GAL or NOS was observed in these cells (Table 2). It is worth mentioning that ZnT3^+^ myenteric neurons were supplied with the thick network of VAChT^+^, LENK^+^, and/or SP^+^ nerve fibers and the moderate number of SOM^+^ and/or VIP^+^ nerve terminals (Fig. 2). Only a small number of NPY^+^ and NOS^+^ nerve fibers and very few CGRP^+^ nerve terminals were observed around ZnT3^+^ myenteric neurons.

### Outer Submucosal Plexus

**Fig. 3:**
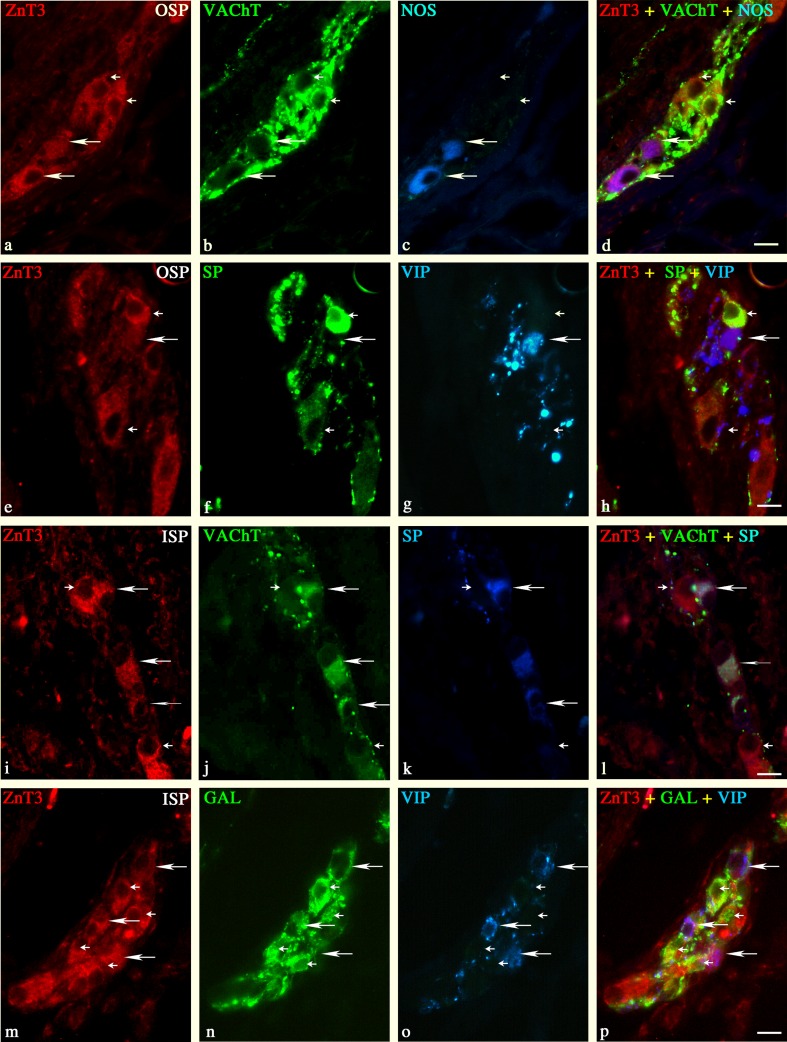
Representative images of ZnT3^+^ neurons located in the duodenal OSP (**a**–**h**) and ISP (**i**–**p**) of the pig. Merged images (**d**, **h**, **l**, **p**) are composites of images taken separately from *red* (**a**, **c**, **i**, **m**), *green* (**b**, **f**, **j**, **n**), and *blue* (**c**, **g**, **k**, **o**) fluorescent channels. *Scale bar* = 25 μm. **a**–**d** ZnT3^+^/VAChT^−^/NOS^+^—*long arrows*, ZnT3^+^/VAChT^+^/NOS^−^—*small arrows*; **e**–**h** ZnT3^+^/SP^−^/VIP^+^—*long arrow*, ZnT3^+^/SP^+^/VIP^−^—*small arrows*; **i**–**l** ZnT3^+/^VAChT^+^/SP^+^—*long arrows*, ZnT3^+^/VAChT^+^/SP^−^—*small arrows*; **m**–**p** ZnT3^+^/GAL^+^/VIP^−^—*long arrows*, ZnT3^+^/GAL^−^/VIP^+^—*small arrows*

The non-cholinergic ZnT3^+^ neurons in the OSP constituted 43.1 ± 5.4 (Table 2). Among these neurons, the most numerous were ZnT3^+^ cells immunoreactive to NOS (19.1 ± 4.8 %; Table 2 and Fig. 3a, c–d). In addition, 10.5 ± 3.1, 4.7 ± 1.6, 3.9 ± 2.5, and 0.4 ± 0.2 % of the non-cholinergic ZnT3^+^ neurons were also immunoreactive to SOM, VIP, SP, and/or LENK, respectively (Table 2, Fig. 3e–h). On the other hand, none of the non-cholinergic ZnT3^+^ neurons was ever immunoreactive to GAL, NPY, or CGRP. The cholinergic ZnT3^+^ neurons in the OSP constituted 56.9 ± 6.4 % (Table 2 and Fig. 3b). The triple-labeling immunofluorescence revealed that 7.4 ± 4.1 % of the cholinergic ZnT3^+^ neurons in the OSP were also immunoreactive to SOM (Table 2). Moreover, approximately 3.7 ± 1.4 and 4.0 ± 1.7 % were also immunoreactive to VIP and SP (Table 2 and Fig. 3e–h). On the other hand, none of the cholinergic ZnT3^+^ neurons was ever immunoreactive to GAL and NOS (Table 2 and Fig. 3d). Both the non-cholinergic and cholinergic ZnT3^+^ neurons were supplied with a thick network of VAChT^+^ or SOM^+^ and a moderate number of SP^+^ or VIP^+^ nerve fibers (Fig. 3d, h). Only single NOS^+^ and LENK^+^ nerve fibers were observed around ZnT3^+^ neurons within the OSP.

### Inner Submucosal Plexus

In the ISP, all ZnT3^+^ neurons were simultaneously cholinergic, i.e., ZnT3^+^/VAChT^+^ (Table 2 and Fig. 3i–j, l). The triple-labeling immunofluorescence revealed that among these neurons, 42.5 ± 1.2 % of cells were also GAL^+^, 36.6 ± 4.2 % were also VIP^+^, 26.2 ± 2.9 were also SP^+^, and 13.2 ± 3.8 coexpressed SOM (Table 2 and Fig. 3k, m–p). In addition, ZnT3^+^ neurons in the ISP were supplied with a very large number of VAChT^+^ nerve terminals and a moderate number of SOM^+^, SP^+^, and/or GAL^+^ nerve fibers (Fig. 3j–l, n–p). On the other hand, only a small number of VIP^+^ nerve fibers and single LENK^+^ and/or NOS^+^ nerve terminals were found around ZnT3^+^ neurons in the ISP.

## Discussion

The present study for the first time demonstrates existence of the ZnT3+ neurons in the ENS of the porcine duodenum. The perikarya immunoreactive to ZnT3 have been found within all types of enteric ganglia, and these results are generally in agreement with previous investigations on the human and porcine large intestine (Gonkowski et al. 2007, 2009). According to the general knowledge about mammalian ENS, the neurons in the MP mainly participate in the regulation of gut motility (Brehmer et al. 1994; Huizinga et al. 2011) whereas the nerve cells within the OSP and ISP are taking part in excretory functions of the digestive tract (Brehmer et al. 2010). Thus, the considerable quantity of ZnT3-expressing cells, above 35 % of all perikarya in each plexus, may suggest that ZnT3 and Zn are important substances within the digestive tract, and they may be engaged at least in some of the gut's functions. Moreover, the previous study on the porcine large intestine (Gonkowski et al. 2007) indicates the participation of ZnT3 and Zn in mechanisms of pathological states within the digestive tract.

Although the precise function(s) of ZnT3 neurons and Zn is currently unknown in the enteric ganglia (this study and Gonkowski et al. 2007, 2009) and in the adrenergic and cholinergic sympathetic neurons of the murine peripheral nervous system (Wang et al. 2001a, 2003; Wang and Dahlstrom 2008), one could expect that ZnT3 and ionic zinc in the porcine duodenum may be, at least in part, implicated as a neurotransmitter/modulatory agent like within the CNS, where the functions of ZnT3 and Zn are better known (Cousins et al. 2006; Danscher et al. 2003; Kim et al. 2000; Palmiter and Huang 2004; Wang et al. 2003; Wenzel et al. 1997). It is generally accepted that ZnT3 plays an important role in the regulation of zinc levels, and it is the key protein in synaptic vesicle zinc transport (Danscher et al. 2003; Wenzel et al. 1997). Previous investigations on the CNS have suggested that ZnT3^+^ and Zn nervous structures may be involved in either the sensory transmission or the efferent control of other neuronal circuits (Danscher et al. 2001, 2003). Both ZnT3 and Zn are primarily present in glutamatergic terminals within the CNS (Danscher et al. 2001). However, Zn is also present in the GABA- and glycine-containing neurons (Danscher et al. 2001; Birinyi et al. 2001; Wang et al. 2001b). In the hippocampus, Zn is co-released with glutamate from ZEN synapses, both spontaneously and with electrical stimulation, where it exerts a strong modulatory effect on *N*-methyl-d-aspartate (NMDA) receptors (Vogt et al. 2000). Moreover, there is some convincing evidence to suggest that Zn may be a potent modulator of various receptors, ion channels, and several transporters in the brain, thereby influencing both excitatory and inhibitory neurotransmission (Betz and Laube 2006; Frederickson et al. 2005; Smart et al. 2004). Excitatory NMDA receptors are directly inhibited by Zn whereas non-NMDA receptors appear relatively unaffected (Smart et al. 2004; Paoletti and Neyton 2007). Because it is concentrated and released at many glutamatergic synapses, Zn is likely to be an endogenous allosteric modulator of the NMDA receptor (Paoletti and Neyton 2007). Inhibitory transmission mediated via GABA_(A)_ receptors can be potentiated via a presynaptic mechanism, influencing transmitter release; however, although some tonic GABAergic inhibition may be suppressed by Zn, most synaptic GABA receptors are unlikely to be modulated directly by this cation (Smart et al. 2004). The glycinergic (inhibitory) transmission in the CNS may also be affected by Zn, thereby causing potentiation (Betz and Laube 2006). Low concentrations of Zn potentiate submaximal glycine-induced currents, whereas higher concentrations cause competitive inhibition (Betz and Laube 2006). A point mutation in the murine Glra1 locus, which selectively suppresses Zn potentiation, generates a phenotype that mimics that of patients with hyperekplexia (hereditary startle disease) and thus is indicative of decreased glycinergic inhibition (Betz and Laube 2006). It is worthy to note that cholinergic receptors in the brain may also be directly up- and downregulated by Zn while various other neurons may be modulated via other types of receptors like opioid or catecholamine receptors, various transporters, and several ion channels, including Ca^2+^, K^+^, or Cl^−^ (Frederickson et al. 2005). These data, taken together, indicate that Zn is a potent modulator of both excitatory and inhibitory neurotransmission within the CNS. Therefore, it is tempting to speculate that it may have a similar role in the ENS. Such mechanism(s) may in part explain why so many excitatory cholinergic and inhibitory nitrergic intestinal neurons coexpress ZnT3 (present study).

Recent studies in the brain and other tissues indicate that ZnT3 and Zn may be implicated not only in neurotransmission but also in many other functions. For example, previous studies on Zn-containing neurons in the CNS suggest that ZnT3 may protect neurons from the cytotoxic action of zinc during pathological processes (Cousins et al. 2003, 2006; Kim et al. 2000; Palmiter and Huang 2004). The number of ZnT3-containing neurons was also elevated in the large intestine during inflammation and axotomy suggesting the existence of similar mechanisms in the ENS (Gonkowski et al. 2007). Observations in neurons suggest that exposure to elevated levels of Zn can be cytotoxic, within minutes (Yokoyama et al. 1986). Several studies in the gastrointestinal tract confirm that oxidant stress induces Zn elevation that can influence pathways of cell death and the balance between necrosis and apoptosis (Cima et al. 2006; Kohler et al. 2009, 2010). Alternatively, extracellular Zn acting via zinc-sensing receptors (ZnR) is able to activate major signaling pathways linked to cellular proliferation and survival (Hershfinkel et al. 2001; Cohen et al. 2012). For example, in the intestines, extracellular Zn, acting through ZnR, regulates intracellular pH and clusterin expression thereby enhancing survival of colonocytes (Cohen et al. 2012). Similar zinc sensors might be present in other tissues, including the neurons. Moreover, extracellular Zn is not the only player in the animal tissues. For example, Ca^2+^ facilitates Zn uptake, and oxidative dysregulation of intracellular Zn could be amplified by dysregulation of intracellular Ca^2+^ homeostasis (Liu et al. 2011). This potential relationship between Zn and Ca^2+^ levels may in part explain the role of Zn and Zn transporters in the intestinal secretion (Liu et al. 2011). Based on the well-recognized role of Ca^2+^ as a second messenger in responses to secretagogues (Caroppo et al. 2001; Perez-Zoghbi et al. 2008), there is a novel concept that Ca^2+^ may be also a second messenger that can match the basolateral demand for Zn with the secretory response to physiologic stimulation (Liu et al. 2011). Similar connections may be also present in other secretory cells including neural, endocrine, and exocrine tissues.

Among the possible ways to elucidate the functions of ZnT3 within the ENS are investigations on co-localization of this peptide with other better known active substances in the same cell bodies. It is generally accepted that each enteric neuron can contain several active substances. Moreover, immunohistochemical investigations on the ENS revealed that each functional class of enteric neurons respectively contains a unique combination of chemical markers (for review, Furness 2012). The present study has shown that ZnT3 in enteric neurons of the porcine duodenum were co-localized with various active substances such as VAChT, NOS, SOM, SP, GAL, or LENK, and the percentage of co-localization was dependent on the type of enteric plexus. Most of the ZnT3^+^ myenteric neurons were also immunoreactive to VAChT and/or NOS. These neurons are one of a number of elements that are responsible for the control of digestive motility (Huizinga et al. 2011), and a subpopulation of myenteric neurons also innervates the submucosal plexuses and/or regulates the secretory functions of the gut (Brehmer et al. 1994). VAChT is the histochemical marker of acetylcholine, and gut motility is controlled by a subpopulation of cholinergic and nitrergic neurons which mediate the respective contraction and relaxation of circular and longitudinal muscle (Brehmer et al. 2006; Boeckxstaens et al. 1993; Furness 2006; Lincoln et al. 1997; Porter et al. 1996, 1997; Wood et al. 1999; Qu et al. 2008). Since the majority of ZnT3^+^ myenteric neurons were also immunoreactive to VAChT and/or NOS, thus ZnT3 and Zn may probably modulate the activity of both neuron populations that act together in coordinated reflexes to facilitate gut smooth muscle contraction (VAChT neurons) and muscle relaxation (NOS neurons), respectively. The neurons of the OSP and ISP innervate the submucosal blood vessels and regulate the secretion and intrinsic sensory pathways of the gut in response to the contents of the lumen (Brehmer et al. 2010). A subpopulation of the OSP neurons can also supply the circular muscle layer of the gut (Scheuermann and Timmermans 1993). More than half of all ZnT3^+^ neurons located in the OSP and all these neurons in the ISP were simultaneously immunoreactive to VAChT. Such a large population of ZnT3^+^/VAChT^+^ neurons indicate that ZnT3 and Zn may be engaged in various excitatory functions in the OSP and ISP including muscle contraction in the muscularis mucosa and the increased activity of mucosal glands (Cooke 2000). VIP was coexpressed in ZnT3^+^ neurons of both submucosal plexuses but especially in the ISP. This is congruent with the fact that submucosal VIP neurons are responsible for increasing secretory activity at mucosal glands in the small intestine (Olsson and Holmgren 2001). GAL was highly coexpressed by ZnT3^+^ neurons in the ISP, while in the OSP, such cells were rare (almost half of ZnT3^+^ neurons in the ISP coexpressed GAL). GAL is often expressed in the ISP by neurons which are engaged in the regulation of the intestinal secretion (Furness 2006) and/or neurotransmitters secretion from other intestinal neurons (Piqueras et al. 2004; Sarnelli et al. 2004). Extensive co-localization of ZnT3 and GAL in the ISP neurons may further indicate that Zn and ZnT3 are involved in these processes of the gut.

In summary, the large number of ZnT3^+^ neurons in the ENS of the porcine duodenum and a broad spectrum of active substances which co-localize with this peptide as well as the previous studies on the porcine digestive tract (Gonkowski et al. 2007, 2009) suggest that ZnT3 is involved in the regulation of various processes in the gut, both in physiology and during pathological processes. By analogy to previous investigations on the central nervous system (Cousins et al. 2006; Danscher et al. 2003; Kim et al. 2000; Palmiter and Huang 2004; Wang et al. 2003; Wenzel et al. 1997), it is likely that ZnT3 is present in enteric neurons, which use zinc as neuromodulator, and/or it has a functional role that protects against the cytotoxic action of zinc. However, further studies are needed to elucidate the exact physiological role of ZnT3 within the enteric nervous system of the porcine GI tract.
